# Draft whole-genome sequence of *Lachancea fermentati* JT139 isolated from Jamestown Island, Virginia

**DOI:** 10.1128/mra.00771-25

**Published:** 2026-02-04

**Authors:** Hanna Masonholder, Kate Lee, Stephen Fong, Grace Lim-Fong

**Affiliations:** 1 Department of Biology, Randolph-Macon College 1342, Ashland, Virginia, USA; 2 Hardywood Park Craft Brewery, Richmond, Virginia, USA; 3 Department of Chemical and Life Science Engineering, Virginia Commonwealth University 6889, Richmond, Virginia, USA; University of California Riverside, Riverside, California, USA

**Keywords:** *Lachancea*, beer, wild yeast

## Abstract

We report the draft whole-genome sequence of *Lachancea fermentati* JT139, a wild yeast isolated from Jamestown Island, Virginia, USA, which was utilized in brewing a commemorative beer. The assembled genome consists of 15 scaffolds totalling 10.2 Mbp, with 42.0% GC content.

## ANNOUNCEMENT

The centuries-long domestication of brewer’s yeast has resulted in strains with fairly uniform fermentation characteristics (1). Seeking to craft beers with unique flavor profiles, we captured undomesticated yeasts on Jamestown Island, where English settlers brewed beer in the 1700s. We reasoned that wild yeasts from surrounding flora likely entered colonial brews and imbued additional flavors. We here describe a yeast species isolated in 2016. Sterile wort in open 75 × 45 × 15 cm containers (approximately 4 L of wort per container) was placed around Jamestown Island (37.2095°N, 76.7787°W) overnight. A portion of the wort (~200 mL) was then transferred to an airlock-fitted flask for a further weeklong incubation at 25°C before plating ~0.5 mL on Universal Beer Agar (BD Difco) and incubating at the same temperature to isolate microbes. Isolates were grown in YPD liquid medium before long-term storage at −80°C. One isolate, JT139, was chosen to brew a beer to celebrate the 400th anniversary of the Jamestown settlement.

DNA was extracted from a colony of JT139 using the Y-PER reagent (2). The internal transcribed spacer region was PCR-amplified (3) and sequenced. *Lachancea fermentati* was identified as the closest match (GenBank accession number MK267548), and the 620 bp sequence was deposited in GenBank (PV700372).

*L. fermentati* JT139 was grown on YPD liquid media at 30°C overnight from a −80°C glycerol freezer stock, after which DNA was extracted following the Hoffman-Winston protocol (4). A library of 400 ng of genomic DNA was then prepared for Oxford Nanopore Technologies (ONT) sequencing using the Rapid Sequencing Kit (SQK-RAD004) without size selection. Sequencing was performed on a MinION Mk1C device using a FLO-MIN106 (R9 Chemistry) flow cell. The run produced 282,247 reads with a read N_50_ of 4,999 bp. Base-calling was performed using MinKNOW 23.04.5 in high-accuracy mode.

Reads were uploaded onto the Galaxy server (https://usegalaxy.org/ [5]) on which most downstream analyses were performed unless noted. Default parameters were used except where otherwise noted. The data set was filtered using Filter FASTQ (Galaxy Version [GV] 1.1.5 [6]) to remove reads shorter than 1,000 bp, and the remaining reads were then trimmed of their ONT adapters with Porechop (GV0.2.4+galaxy0 [7]). These reads were assembled using Flye (GV2.9.3+galaxy0 [8)) with an estimated genome size of 12 Mbp based on the reference genome of *L. fermentati*. The assembly was then polished using Medaka Consensus Pipeline (GV1.7.2+galaxy1 [9]) under the r941_min_hac_g507 model. Gfastats (GV1.3.6+galaxy0 [10]) was used to generate genome assembly statistics, and the BUSCO (GV5.5.0+galaxy0 [11]) was used to assess genome assembly completeness against the Saccharomycetes_odb10 marker set. The average nucleotide identity of the assembled genome was then compared to that of *L. fermentati* CBS 6772 (European Nucleotide Archive accession number GCA_900074765) using the web service https://www.ezbiocloud.net/tools/ani (12).

The 233,869 filtered and trimmed reads had a median quality of 12.7 and a N_50_ of 8,133 bp. Assembly in Flye and polishing in Medaka resulted in the genome assembly characteristics detailed in Table 1. The genome assembly was estimated by BUSCO to be 98.9% complete.

**TABLE 1:** Genome assembly features of JT139

Feature	Value
Number of scaffolds	15
Total length of scaffolds (bp)	10,181,034
Scaffold N_50_ (bp)	1,196,665
GC content (%)	42.0
Estimated sequencing coverage	133×
Average nucleotide identity compared to *L. fermentati* CBS 6772 (%)	92.8

## Data Availability

The Lachancea fermentati JT139 strain was deposited into the Phaff Yeast Culture Collection. Data were deposited at GenBank under SRA accession number SRX29608038, and the genome assembly was submitted under GCA_053726775.1.
